# The utility of single-balloon enteroscopy for the diagnosis and management of small bowel disorders according to their clinical manifestations: a retrospective review

**DOI:** 10.1186/1471-230X-13-103

**Published:** 2013-06-22

**Authors:** Varayu Prachayakul, Morakod Deesomsak, Pitulak Aswakul, Somchai Leelakusolvong

**Affiliations:** 1 Department of Internal Medicine, Faculty of Medicine, Division of Gastroenterology, Siriraj Hospital, Mahidol University, Bangkok, Thailand; 2 Siriraj GI Endoscopy Center, Siriraj Hospital, Mahidol University, Bangkok, Thailand; 3 Liver and Digestive Institute, Samitivej Sukhumvit Hospital, Bangkok, Thailand

**Keywords:** Single balloon, Enteroscopy, Performance, Therapeutic impacts, Indications, Complications

## Abstract

**Background:**

The advent of double-balloon enteroscopy has enabled more accurate diagnosis and treatment of small bowel disorders. Single-balloon enteroscopy permits visualization of the entire small intestine less often than does double-balloon enteroscopy. However, the relative clinical advantages of the 2 methods remain controversial. This study therefore aimed to identify the indications for and therapeutic impact of performing single-balloon enteroscopy.

**Methods:**

We retrospectively reviewed prospectively collected data from adults who underwent single-balloon enteroscopy from January 2007 through November 2011 and analyzed their baseline characteristics, endoscopic findings, pathological diagnoses, and clinical outcomes.

**Results:**

A total of 145 procedures were performed in 116 patients with a mean age of 58.1 ± 17.7 years (range, 18–89 years). The most common indications for performing single-balloon enteroscopy were overt gastrointestinal (GI) bleeding, chronic diarrhea, and occult GI bleeding, accounting for 57.9%, 12.4%, and 9.7% of the patients, respectively. The area of interest was achieved in 80.7% of the cases, with a 5.5% rate of technical failure. An overall positive finding was detected in 65.5% of the cases, of which 33.8% were ulcers and erosions; 8.3%, masses; and 3.4%, angiodysplasia. The diagnostic yields were 42.9%, 52.4%, 78.6%, 50.0%, and 25.0% for patients with overt GI bleeding, occult GI bleeding, abdominal pain, chronic diarrhea, and abnormal imaging results, respectively. Therapeutic procedures were performed in 11% of patients with GI bleeding and achieved a therapeutic yield of 14.6% with a minor complication rate of 11.7%.

**Conclusions:**

Single-balloon enteroscopy was effective for the diagnosis and treatment of small bowel disorders, especially in patients who presented with abdominal pain, GI bleeding, or focal abnormalities on imaging scans.

## Background

The inaccessibility of the small intestine has traditionally made it the “final frontier” for gastroenterologists. However, the past decade has seen the development of 4 nonsurgical, push-and-pull flexible endoscopy techniques: balloon-assisted enteroscopy using 2 balloons (double-balloon enteroscopy [DBE]) or 1 balloon (single-balloon enteroscopy [SBE]), balloon-guided enteroscopy (BGE), and the newly developed spiral enteroscopy (SE), which pleats the small bowel via rotation of an overtube. DBE, the first balloon-assisted enteroscopy technique developed, was introduced in 2001 [1]. It requires a significant learning curve and a great deal of experience on the part of the endoscopists who perform the procedure, and the procedure itself is cumbersome. To overcome these limitations, SBE was developed by removing the balloon on the enteroscope and using the hook/suction-and-pull technique in its place; the resulting endoscope, equipped with only 1 balloon, expected to be easier to handle. There have been several reports of significant differences in the total endoscopy rate between DBE and SBE. DBE achieves total enteroscopy at a 3-fold higher rate than SBE or SE [2-5]. However, the contributions of total enteroscopy to the diagnostic yield and the management of small bowel disease remain controversial [6,7].

SBE was developed by Olympus, Inc. (Tokyo, Japan) in 2006, and the insertion technique was notably well-established [8]. However, only limited data on SBE are available. To our knowledge, only a few published studies have compared SBE and DBE directly. Although the insertion depth is smaller for SBE than for DBE, the diagnostic and therapeutic yields of these techniques do not greatly differ. Therefore, the insertion depth or examination of the entire small intestine (total enteroscopy) might not contribute to the diagnostic and therapeutic yields [6,9-11]. The primary objective of this retrospective study was to evaluate the clinical utility of SBE in patients with suspected small bowel disease according to the different indications for performing the procedure.

## Methods

This retrospective review was performed at a large endoscopy center within a university-based hospital using data from January 2007 through November 2011. The present study was approved by the Siriraj Institutional Review Board (SIRB). Most of the procedures were performed by a few dedicated gastroenterologists, each of whom had performed more than 30 SBEs. The electronic and paper-based medical records were reviewed. The patients’ baseline characteristics such as their demographic data, indications for enteroscopy, procedure details, anesthetic methods, immediate and short-term complications, pathological reports, and treatments, were analyzed. The indications for performing enteroscopy included overt gastrointestinal (GI) bleeding; occult GI bleeding; anemia; abnormalities on computed tomography (CT) or magnetic resonance imaging (MRI) scans or in the results of small bowel barium studies (simple barium study of the small intestine, not enteroclysis); abdominal pain; and chronic diarrhea. Overt bleeding was defined as obvious bloody content on initial presentation with negative esophagogastroduodenoscopy and colonoscopy results. Occult bleeding was defined as a positive guaiac fecal occult blood test result without obvious bleeding. Anemia was defined as a low hemoglobin level (<13 mg/dL for men or <12 mg/dL for women) without evidence of overt or occult GI bleeding. Enteroscopy was performed in patients with abdominal pain if the etiology of the pain was unclear; this included cases of suspected chronic inflammatory bowel disease or small intestinal infection. Chronic diarrhea was defined as diarrhea lasting for >4 weeks. Informed consent was obtained from all the patients. All procedures were performed in the endoscopic suite with full anesthetic monitoring. SBE (SIF-Q180, Olympus Optical, Tokyo, Japan) was performed with the patient under total intravenous anesthesia (TIVA) or general anesthesia (GA) in all cases. The insertion depth is not measured in centimeters at our institute; therefore, in our study, the performing endoscopist estimated the insertion depth at the end of the procedure in terms of the region to which the endoscope was inserted, such as the proximal jejunum, mid-jejunum, distal jejunum, proximal ileum, mid-ileum, or distal ileum. Total enteroscopy refers to bidirectional endoscopy in which the enteroscope is passed during the second procedure through the site marked by tattooing during the first procedure. The procedure time extended from the time at which the scope was passed through the mouth until the time at which it was completely withdrawn from the mouth (antegrade route) or from the time at which the scope was passed through the anus to the time at which it was completely withdrawn from the anus (retrograde route). A procedure was considered a failure when it was terminated because the endoscopist could not advance the enteroscope deeper than the proximal jejunum owing to altered or abnormal anatomy not identified before the procedure. The areas of interest achieved were recorded. The diagnostic yield was defined as the percentage of procedures that produced either a definitive diagnosis or findings that could explain the clinical symptoms. Clinical success was defined as the effectiveness of enteroscopy at obtaining a definitive diagnosis and/or treating lesions or, in cases of overt GI bleeding, at preventing further bleeding until the last follow-up visit. All of the data were recorded and analyzed using SPSS version 13.0. The results were summarized using standard methods. The parametric data were reported as percentages. The nonparametric continuous data were expressed as the mean ± SD. The differences between the nonparametric unpaired continuous data were analyzed using the chi-square or Fischer’s exact test. A *p* value of less than 0.05 was defined as significant.

## Results

A total of 145 consecutive SBE procedures were performed in the 116 patients who included in our study. The mean age of the patients was 58.1 ± 17.7 years (range, 18–89 years), and the male-to-female ratio was 1:1. Of the patients, 22% underwent a retrograde procedure, whereas 77% underwent an antegrade procedure. Only 1 patient underwent a bidirectional procedure in a single session, although another 15 bidirectional procedures were performed sequentially. Of these 15, 8 were completed within 7 days after the first procedure. The average time between the 2 procedures was 11.3 days. The indications for enteroscopy were overt GI bleeding, occult GI bleeding, chronic diarrhea, abdominal pain, and abnormal imaging results in 57.9%, 22.1%, 12.4%, 8.3%, and 5.5% of procedures, respectively. All patients underwent extensive evaluations including video capsule enteroscopy (VCE) in 46.2% of the patients and imaging examinations such as CT, MRI, or barium studies in 23.4% of the patients, before undergoing SBE. Fluoroscopy was used in 71.7% of the procedures. Of the patients, 12.4%, 55.8%, 33.1%, and 0.7% were classified as American Society of Anesthesiologists (ASA) class I, II, III, and IV, respectively. During the procedure, 40% of the patients were maintained under TIVA and 59.3% under GA. Total enteroscopy was achieved in 1 (6.23%) of 16 attempts. Eight procedures (5.5%) were considered failures. The mean ± SD procedure duration was 82.4 ± 43.2 min for all procedures, 81.8 ± 41.2 min for antegrade procedures, and 78.3 ± 34.7 min for retrograde procedures. The areas of interest were achieved in 80.7% of the patients, and positive findings were detected in 60.0%. However, the positive findings in this study were further classified on the basis of their relevance to the clinical manifestations as definitive diagnoses, associated positive findings, and incidental diagnoses. A definitive diagnosis was obtained in 32.4% of the patients and an overall diagnosis (either a definitive diagnosis or an associated positive finding) in 47.6%. Therapeutic interventions were performed in only 16 patients (11.0%). No serious complications occurred in the cases reviewed for this study.

The most common enteroscopic findings obtained via both the antegrade and retrograde routes of insertion were erosions and ulcers, including angiodysplasia. The significant findings obtained via the antegrade route were Dieulafoy’s lesions, varices, polyps, and masses, as shown in Table 1.

**Table 1:** Endoscopic findings according to the route of insertion

**Finding**	**Total**	**Antegrade**	**Retrograde**
	**(n = 145)**	**(n = 112)**	**(n = 34)**
	**n (%)**	**n (%)**	**n (%)**
**Angiodysplasia**	5 (3.4)	4 (3.6)	1 (2.9)
**Ulcer**	26 (17.9)	17 (15.2)	9 (26.4)
**Erosion**	23 (15.9)	21 (18.8)	2 (5.9)
**Stricture**	6 (4.1)	4 (3.6)	2 (5.9)
**Polyp**	8 (5.5)	8 (7.1)	0
**Mass**	12 (8.3)	12 (10.7)	0
**Irregular/erythematous mucosa**	26 (17.9)	25 (22.3)	1 (2.9)
**Blood stain**	5 (3.4)	4 (3.6)	1 (2.9)
**Lipoma**	1 (0.7)	1 (0.9)	0
**Villous atrophy**	7 (9.3)	7 (6.3)	0
**Diverticulum**	4 (2.8)	4 (3.6)	0
**Dieulafoy’s lesion**	5 (3.4)	5 (4.5)	0
**Varix**	2 (1.4)	2 (1.8)	0
**Hemangioma**	1 (0.7)	1 (0.9)	0
**Lymphangiectasia**	14 (9.7)	14 (12.5)	0

The patients’ demographic data, enteroscopic procedure details, and positive findings were analyzed according to the clinical manifestations, as shown in Table 2. The diagnostic yields were 42.9%, 52.4%, 78.6%, 50.0%, and 25.0% for patients with overt GI bleeding, occult GI bleeding, abdominal pain, chronic diarrhea, and abnormalities on imaging scans, respectively.

**Table 2:** Demographic data, details of the endoscopic procedures, endoscopic findings, and clinical outcomes according to the indication for small bowel enteroscopy

**Indication**	**Overt GI bleeding**	**Occult GI bleeding**	**Abdominal pain**	**Chronic diarrhea**	**Abnormalities on imaging scans**
**Details**	**(n = 84)**	**(n = 21)**	**(n = 14)**	**(n = 18)**	**(n = 8)**
	**n (%)**	**n (%)**	**n (%)**	**n (%)**	**n (%)**
**Age (years)**	61.8 ± 18.0	81.0 ± 18.3	50.4 ± 12.7	47.5 ± 14.5	55.7 ± 15.5
**Sex: male**	51 (60.7)	6 (28.6)	9 (64.5)	5 (27.8)	4 (50.0)
**Route: antegrade**	60 (71.4)	18 (85.7)	11 (78.6)	16 (88.9)	7 (87.5)
**Areas of interest achieved**	61 (72.6)	20.0 (95.2)	11 (78.6)	18 (100)	7 (87.5)
**Findings**					
**Negative**	37 (44.0)	7 (33.3)	3 (21.4)	6 (33.3)	5 (62.5)
**Positive**	48 (56.0)	14 (66.7)	11 (78.6)	12 (66.7)	3 (37.5)
**Erosions**	13 (15.5)	3 (14.3)	11 (78.6)	3 (16.7)	1 (12.5)
**Ulcers**	14 (16.7)	3 (14.3)	6 (42.9)	3 (16.7)	0 (0.0)
**Stricture**	0 (0.0)	2 (9.5)	4 (28.6)	0 (0.0)	0 (0.0)
**Angiodysplasia**	4 (4.8)	1 (4.8)	0 (0.0)	0 (0.0)	0 (0.0)
**Polyp**	1 (1.2)	4 (19.0)	0 (0.0)	3 (16.7)	0 (0.0)
**Mass**	8 (9.5)	2 (9.5)	1 (7.1)	0 (0.0)	1 (12.5)
**Non-specific erythematous mucosa**	5 (6.0)	2 (9.5)	9 (64.2)	7 (38.9)	1 (12.5)
**Blood stain**	4 (4.8)	0 (0.0)	1 (7.1)	0 (0.0)	0 (0.0)
**Atrophic villi**	0 (0.0)	0 (0.0)	1 (7.1)	0 (0.0)	0 (0.0)
**Dieulafoy’s lesion**	5 (6.0)	0 (0.0)	0 (0.0)	0 (0.0)	0 (0.0)
**Diverticulum**	4 (4.8)	0 (0.0)	0 (0.0)	0 (0.0)	0 (0.0)
**Varix**	2 (2.4)	0 (0.0)	0 (0.0)	0 (0.0)	0 (0.0)
**Hemangioma**	1 (1.2)	0 (0.0)	0 (0.0)	0 (0.0)	0 (0.0)
**Lymphangiectasia**	7 (8.3)	2 (9.5)	3 (21.4)	1 (5.6)	1 (12.5)
**Failure**	7 (8.3)	0 (0.0)	0 (0.0)	0 (0.0)	1 (12.5)
**Therapeutic interventions performed**	11 (13.1)	5 (23.8)	0 (0.0)	0 (0.0)	0 (0.0)
**Clinical conclusion**	37 (44.0)	7 (33.3)	3 (21.4)	6 (33.3)	5 (62.5)
**Negative finding**					
**Incidental finding**	11 (13.1)	3 (14.3)	3 (21.4)	3 (16.7)	1 (12.5)
**Associated positive finding**	11 (13.1)	1 (4.8)	4 (28.6)	5 (27.8)	1 (12.5)
**Definitive diagnosis**	25 (29.8)	10 (47.6)	7 (50.0)	4 (22.2)	1 (12.5)
**Diagnostic yield**	36 (42.9)	11 (52.4)	13 (78.6)	8 (50.0)	2 (25.0)
**Clinical success**	35 (41.7)	11 (52.4)	10 (71.4)	7 (38.9)	3 (37.5)
**Minor complications**	9 (10.5)	3 (14.3)	1 (7.1)	0 (0.0)	2 (25.0)
**Time to enteroscopy (days)**	49.3	N/A	N/A	N/A	N/A
**VCE done**	51 (60.7)	11 (52.4)	2 (14.3)	3 (16.7)	0 (0.0)

Abbreviations: *GI* Gastrointestinal, *VCE* Video capsule enteroscopy, *N/A* Not available.

### Findings

The most common indication (72.4% of procedures) for performing enteroscopy in this study was obscure bleeding (both overt and occult bleeding). The enteroscopic findings are shown in Table 2. The most common endoscopic findings obtained in this study were ulcers and erosions (33.8%), and the second-most common finding was nonspecific changes such as mucosal irregularity or erythematous mucosa. Angiodysplasia was detected in only 3.4% of the patients in this study. Only 67 patients in this study underwent VCE. The enteroscopic finding was consistent with the VCE finding in 33 (48.5%) of 67patients.

### Overt GI bleeding

A total of 84 patients underwent SBE for investigation of overt GI bleeding. The most common enteroscopic findings were erosions and ulcers (32.2%), followed by vascular lesions (13.2%) (consisting of Dieulafoy’s lesions [6.0%], angiodysplasia [4.8%], and varices [2.4%]) and tumors (9.5%). The diagnostic yield of SBE for overt GI bleeding was 42.9%. The definitive causes of overt GI bleeding remained unidentified in approximately 57.1% of the cases. However, we followed up the patients in this subgroup and found that these patients experienced no recurrent bleeding during the mean follow-up period of 46.15 weeks. Therapeutic interventions were performed during 11 procedures and were successful in all but 1 patient (who experienced recurrent bleeding). The outcomes in this group are also shown in Table 2 The clinical success rate of the procedures in this group was low (41.7%); as erosions and ulcers were the most common indications for performing the procedures, we suspected that the low success rate might have been because of healing of the lesions between initial presentation and endoscopy. Therefore, we identified the time to enteroscopy (defined as the interval from the onset of clinical signs to the performance of the procedure), which was 49.3 ± 104.9 days overall, and compared the mean time to enteroscopy between the patients in which SBE was clinically successful and those in which it was not. The time to enteroscopy was 75.9 ± 25.6 days for the first group but 29.5 ± 6.6 days in the second group. However, this difference was not statistically significant (*p* = 0.08). Both VCE and SBE were performed in 60.7% of the patients. The enteroscopic findings were consistent with the VCE findings in 73.3% of the patients with positive enteroscopic indings and in 36.1% of the patients with negative enteroscopic findings (data not shown).

### Occult GI bleeding

Twenty-one procedures were performed to investigate occult GI bleeding. The mean age of these patients was 81.0 ± 18.3 years, which was higher than in the other groups. The most common enteroscopic findings were erosions and ulcers (28.6%), followed by tumors (9.5%) and angiodysplasia (4.8%). No Dieulafoy’s lesions were found in this group. The areas of interest were achieved in approximately 95.2% of the procedures The diagnostic yield of SBE for occult GI bleeding was 52.4%. However, the definitive cause of occult GI bleeding remained unidentified in approximately 47.6% of the patients. Therapeutic interventions were performed during 5 procedures and were successful in all cases. The clinical success rate in this group was 50%. Both VCE and SBE were performed in 52.4% of the patients. The enteroscopic findings were consistent with the VCE findings in all of the patients with positive enteroscopic findings and in 12.5% of the patients with negative enteroscopic findings (data not shown).

### Abnormalities on imaging studies

The diagnostic yield of SBE was 25% in the patients who presented with abnormalities on imaging scans. The most common enteroscopic findings were erosions (12.5%) and tumors (12.5%). However, the definitive causes of the abnormalities on the imaging scans remained unidentified in approximately 75% of the patients. Of the 8 patients, 4 who had diffuse or long-segment thickening of the small bowel on CT scans had negative SBE findings (data not shown), whereas 1 who had a bowel mass and 1 who had a focal area of bowel wall thickening on imaging scans had positive SBE findings.

### Abdominal pain

Of the 14 patients who presented with abdominal pain, 7 underwent imaging studies. The diagnostic yield of SBE was 78.6%, and the most common findings were erosions (78.6%) and ulcers (42.9%), followed by strictures (28.6%), of which 5 of 7 patients were diagnosed with Crohn’s disease (data not shown). A tumor was identified as the cause of the abdominal pain in only 1 patient (7.1%) in this group.

### Chronic diarrhea

A diagnostic yield of 50.0% was obtained in 9 of 18 patients. The most common enteroscopic finding was nonspecific changes (38.9%), followed by erosions and ulcers (33.4%). Mass lesions were not the cause in this group. The definitive diagnoses, as determined by histopathological examination, were eosinophilic enteritis, lymphoma, Crohn’s disease, and *Capillaria philippinensis* infestation.

### Therapeutic interventions

Therapeutic interventions were performed in 16 patients (11.0%) with overt or occult GI bleeding (11 procedures in patients with overt GI bleeding and 5 in patients with occult GI bleeding). The therapeutic interventions involved epinephrine injection in 10 patients, hemostatic clip application in 9, argon plasma coagulation (APC) in 9, polypectomy in 4, and Histoacryl® injection in 1. The technical success rate was 100%, with an overall clinical success rate of 93.7%. The therapeutic intervention failed only in 1 patient. That patient had undergone pylorus-preserving pancreaticoduodenectomy and presented with overt GI bleeding a few days after surgery. Active bleeding from an anastomotic site was noted during SBE and was initially stopped by APC. However, the patient experienced recurrent bleeding 1 day later and was finally treated surgically.

### Complications

**Figure 1:**
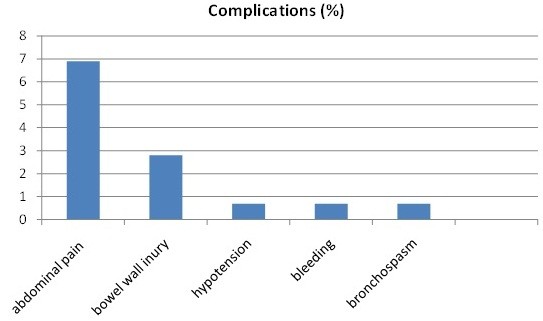
Complications of single balloon enteroscopy.

Complications were reported in 11.7% of the cases in the present study. All were classified as minor complications, as depicted in Figure 1, and most involved only abdominal discomfort and minimal small bowel mucosal trauma, allowing the patients to be discharged on the same day. Neither perforation nor acute pancreatitis was observed in this study.

## Discussion

One-third of the patients in this study were aged >70 years. All of the patients tolerated the procedures well without any significant complications. These findings suggest that SBE is a safe and effective procedure even in elderly patients. Most of the SBE procedures performed in the present study were performed in an antegrade fashion. The failure rate of SBE in this study was 5.5%, which is lower than the 10% failure rate reported by Upchurch et al. [3]. The rate of total enteroscopy in this study was 6.2%, similar to the approximately 0–22% range for SBE reported elsewhere [9-13]. However, these numbers are much lower than the 18–66% rate of total enteroscopy in many reports using DBE. Many experts in balloon-assisted enteroscopy believe that the success of this method depends on the technique used during the pull phase of enteroscopy. However, the area of interest was achieved in approximately 80.7% of procedures, and these procedures yielded an overall diagnosis in approximately 42.8% of cases, which is similar to the rates of 37–61% reported elsewhere for SBE [3,5,10,11,13,14]. In addition, this result was comparable with those of DBE studies that reported diagnostic yields of approximately 43–72% [5,11]. However, some question remains as to whether the technically appreciated end point of complete small bowel visualization is preferable to the more clinically relevant end point of diagnostic yield. Therefore, the utility of total enteroscopy remains unknown at this time and requires further study. The therapeutic yield was lower in this study than in other studies from Europe and America [3-6] that reported therapeutic yields as high as 24–42%, with a bleeding recurrence rate of 20%. However, some of the procedures done in these Western studies, including biopsy and endoscopic tattooing, might have greatly affected the total therapeutic procedure and yield. Most of the patients who underwent therapeutic treatment in the present study, all of whom presented with GI bleeding, achieved initial hemostasis. Therefore, the therapeutic yield for GI bleeding in this study would be 14.6%.

The aim of the present study was to determine the performance of SBE according to the patients’ clinical manifestations. The most common indication for performing enteroscopy in the present study was GI bleeding, as in previous studies. Moreover, the highest diagnostic yield (78.6%) was found in the patients who presented with abdominal pain associated with inflammatory bowel diseases and mass lesions. The diagnostic yield was only 42.9% in the overt GI bleeding group and 54.0% in the occult GI bleeding group. These results were not consistent with those of previous Western studies, which reported diagnostic yields of as high as 78% in patients presenting with GI bleeding [4,5]. In addition, the most common cause of bleeding was erosions and ulcers; this was similar to the findings of other Asian studies but different from those of the Western studies, in which angiodysplasia was the most common finding. Therefore, we hypothesized that the difference in diagnostic yield might be related to the different etiologies of GI bleeding. We suspect that a prolonged time interval between clinical presentation and enteroscopy, i.e., time to enteroscopy, could produce false-negative findings. Therefore, we determined the time to enteroscopy in this study, which was 49.3 ± 104.9 days overall. The reason for this prolonged time to enteroscopy was related to our health system. The patients who were referred for SBE in our hospital (a university-based tertiary care center located in the central part of Thailand) came from all over the country. In general, it takes some time to arrange for such referrals arrangement. However, in the patients with overt GI bleeding, the time interval between the onset of bleeding and the day on which enteroscopy was performed did not differ significantly according to whether a definitive diagnosis was achieved (*p* = 0.08). Therefore, the time to enteroscopy might not be directly related to the diagnostic yield. The previously mentioned hypothesis would also suggest that the low diagnostic rate in this study might be related to the etiology of the bleeding itself, which was most frequently found to be erosions and ulcers. However, the time to enteroscopy in this study was rather prolonged, which may have allowed the lesions to heal and disappear. Furthermore, the therapeutic interventions performed in the GI bleeding groups showed very high success rates. Therefore, patients who present with overt or occult GI bleeding with concurrent blood loss should undergo enteroscopy rather than other modalities, as the diagnostic yield and therapeutic impact are equal to those of VCE. Enteroscopy rather than VCE should be the initial procedure particularly for patients who present with overt GI bleeding, which carries greater potential for therapeutic intervention. The etiology of GI bleeding is also a source of discrepancy. Angiodysplasia has been reported as the most common lesion found in many Western studies, occurring in 6–43% of all cases [8-15]. However, we detected this lesion in only 3.4% of the cases, similarly to many other Asian studies. In our opinion, this may be because of the difference in ethnicity. Dieulafoy’s lesion was reported to be the source of overt GI bleeding in 3.5% of the cases in an Australian study [16]; most of the lesions were located in the proximal jejunum, with a 20% bleeding recurrence rate during 12.5 months of follow up. The present study found Dieulafoy’s lesions as the cause of the GI bleeding in 5 cases (6%); all of these lesions were located in the mid- to distal jejunum, and all associated therapeutic procedures were successful without any recurrence of bleeding. Half of the mass lesions found in this study were GI stromal tumors; lymphoma accounted for one-third of the patients, and only 1 case of intestinal adenocarcinoma was found. Most of the lesions were located in the jejunum. Our results were comparable to those of a meta-analysis of the performance of DBE by Xin L et al. in 2011 [17]. In that study, the most common indication indication for enteroscopy was suspected mid-GI bleeding (62.5%), followed by symptoms/signs only (7.9%), small-bowel obstruction (5.8%), and Crohn’s disease (5.8%). The pooled detection rates were 68.1%, 68.0%, 53.6%, 63.4%, and 85.8% for overall, suspected mid-GI bleeding, symptoms/signs only, Crohn’s disease, and small-bowel obstruction, respectively. Inflammatory lesions (37.6%) and vascular lesions (65.9%) were the most common findings in patients with suspected mid-GI bleeding in Eastern and Western countries, respectively. The pooled rate of total enteroscopy by combined or antegrade-only approaches was 44.0%. GI bleeding was also the most frequent indication in the present study, but our detection rate for GI bleeding was lower. However, the causes of bleeding in this study were predominantly inflammatory rather than vascular lesions, similar to those in other reports from Eastern counties. The rate of total enteroscopy in this study was very low.

In patients with chronic diarrhea, a definitive diagnosis was obtained in only 22% of the cases with diffuse histopathological signs. Therefore, enteroscopy might not be effective in this diagnostic group. The highest diagnostic yield in the present study was for the patients who presented with abdominal pain. Most of these patients were diagnosed with Crohn’s disease. In the group with abnormal imaging study findings, enteroscopy was more effective in the patients with focal lesions than those with diffuse lesions such as small bowel thickening. The SBE findings were negative in most of the patients with diffuse small bowel thickening; therefore, other imaging methods such as VCE might be more useful in such cases. The major complications reported previously, such as perforation and acute pancreatitis, were not observed in this series; only minor complications were detected. The limitations of the present study include the retrospective study design, which might have allowed the inclusion of inaccurate procedure data, such as the insertion depth estimates. Another limitation is the small population in some groups especially those with abnormalities on imaging studies, chronic diarrhea, and chronic abdominal pain, which do not provide adequate data for comparative analysis. Therefore, a prospective study design would be appropriate for future research to validate our findings.

## Conclusions

The data obtained from this series are comparable to the results previously reported for DBE and demonstrate that SBE is a safe and effective procedure for imaging and performing therapeutic interventions in the small bowel. The clinical manifestations for which SBE provided good diagnostic yields were chronic abdominal pain, occult/overt GI bleeding, and focal abnormalities on imaging scans.

## Competing interests

Varayu Prachayakul, Morakod Deesomsak, Pitulak Aswakul, and Somchai Leelakusolvong have no conflicts of interest or financial ties to disclose.

## Authors’ contributions

VP developed the concept; PA and MD contributed to acquisition of data; VP, PA, and SL revised the paper for important intellectual content; VP and PA wrote the paper. All authors read and approved the final manuscript.

## Pre-publication history

The pre-publication history for this paper can be accessed here:

http://www.biomedcentral.com/1471-230X/13/103/prepub
